# Social and structural factors associated with depression and suicidality among men who have sex with men and transgender women in Nepal

**DOI:** 10.1186/s12888-021-03477-8

**Published:** 2021-09-29

**Authors:** Sanna Storm, Keshab Deuba, Rachana Shrestha, Lok Raj Pandey, Deepak Dahal, Madan Kumar Shrestha, Tara Nath Pokhrel, Gaetano Marrone

**Affiliations:** 1 Department of Global Public Health, Karolinska Institutet, Stockholm, Sweden; 2 National Centre for AIDS and STD Control/Global Fund Programs, Kathmandu, Nepal; 3 Public Health and Environment Research Centre, Lalitpur, Nepal; 4 National Centre for AIDS and STD Control, Ministry of Health and Population, Kathmandu, Nepal; 5 Family Welfare Division, Department of Health Services, Ministry of Health and Population, Kathmandu, Nepal

**Keywords:** Sexual and gender minorities, MSM, TG, Discrimination, Depression, Suicidality, Low-income country

## Abstract

**Introduction:**

The prevalence of depression and suicidality is high among men who have sex with men (MSM) and transgender women (TGW) worldwide. Stigma and discrimination are likely contributing factors. More research is needed in low-income, non-English speaking countries to assess the effects of social and structural factors on depression and suicidality among MSM and TGW.

**Methods:**

Nepalese MSM and TGW (*n* = 340) were recruited using a respondent-driven sampling design and filled out a survey questionnaire. The outcomes were depression and suicidality. Data was analyzed using bivariate and multivariable logistic regression.

**Results:**

More than half of the participants (59%) suffered from depression. Severe depression was more common among TGW compared to MSM (41 and 20%, respectively). When it comes to suicidality, TGW had higher lifetime prevalence of suicidal thoughts compared to MSM (32 and 5%, respectively). Depression was positively associated with sex work both for MSM (AOR: 7.9; 95% CI 3.4–18.2) and TGW (AOR: 6.5; 95% CI: 2.3–18.2). MSM who were evicted by family had high odds of suicidal thoughts (AOR: 6.2; 95% CI: 1.3–28.8). For TGW, suicidality was associated with being cheated and threatened (AOR: 3.9; 95% CI: 1.2–12.5) and having forced to marry a female (AOR: 2.2; 95% CI 1.1–5.1).

**Conclusions:**

Nepalese MSM and TGW suffer from a high degree of mental and psychosocial health issues. Future studies should focus on intervention research and on collecting data from a larger variety of gender and sexual minorities.

**Supplementary Information:**

The online version contains supplementary material available at 10.1186/s12888-021-03477-8.

## Introduction

Gender and sexual minorities are a group of individuals “whose sexual orientation or gender identity and reproductive development is considered outside cultural, societal, or physiological norms” [1]. Two groups that represent gender and sexual minorities are men who have sex with men (MSM) and transgender (TG) individuals. TG describes individuals who were assigned female or male sex at birth but whose gender identity does not match the assigned gender [2]. Stigma towards these groups common and a major cause of negative health outcomes [3, 4]. Much of the research on gender and sexual minorities to this day has been conducted in high-income, English speaking countries and focused on physical health [5]. In recent years, growing evidence suggests similar results in studies conducted in low- and middle income countries in Asia [6–9]. In this study, we have focused on mental health aspects among MSM and TG women (TGW) in Nepal.

Compared to heterosexual individuals, sexual minorities are at a greater risk of suffering from psychosocial health problems including depression, suicidality, anxiety and substance abuse [10–12]. In Europe and North America, researchers have shown an association between non-heterosexual identity and mental health issues regardless of age, geographical location and which non-heterosexual orientation a person identifies with [10, 11, 13–16]. When it comes to underlying reasons, a review article from 2018 comprising 35 American studies on depression among sexual minority youth identified several risk factors for depression, such as family rejection, experiences of negative social interactions and abuse, but also protective factors, such as self-esteem [13]. A review of 26 studies revealed that TG identity, as compared to a cis-gender (non-TG) identity, is also associated with a higher prevalence and rates of suicide [15]. The rate of suicidal thoughts amongst TG varied from 10 to 81% in these studies, and attempted suicide rates varied from 10 to 52%.

The findings from Europe and North America on negative mental health aspects among gender and sexual minorities also appear in Southeast Asia [17–20]. According to United Nations Development Programme (UNDP) and Asia Pacific Transgender Network (APTN), discrimination is common in the fields of education, employment and health services as is physical, sexual and psychological abuse of the gender and sexual minorities [2, 21]. In addition, the limited legal rights of these minorities might worsen their mental health [22]. In fact, in half of the Asian countries homosexuality is still illegal [2, 22]. Laws such as public nuisance, criminalization of sex work and prohibition of cross-dressing are frequently used to target transgender individuals as reported by UNDP and APTN [2].

As a consequence of discrimination and lack of legal assistance, many minorities hide their sexual identity [19, 20]. Appearing different than society’s norm is a source of stigma, leading many TGW to choose in gender conforming clothing [20]. As reported by APTN and UNDP, discrimination and stigma are common on the job market, which can lead to unemployment [23]. This, in turn, sometimes leaves sex work as the only source of income, further increasing the stigma [19]. According to a Vietnamese study on 451 MSM, these stigmas might explain why they tend to suffer from increased rates of depression [18]. In the study, as many as 68% of the participants had clinically significant depression.

Although discrimination against minorities is common in Southeast Asia, Nepal can be considered an outlier because Nepal legally recognizes the third gender and the constitution encourages equal rights [21, 24]. In spite of this, gender and sexual minorities still face challenges in their day-to-day life [25, 26]. The problem in Nepal seems be centered on social stigma. Family members, neighbors and other community members try to influence life choices of gender and sexual minorities towards a heteronormative way of living [25]. This discourages them from openly showing their sexual orientation or gender identity [25]. Consequently, homosexual men often marry females [2, 27, 28]. Furthermore, social stigma is also a source of negative feelings, having to be untruthful to family and friends when hiding one’s true sexual identity [27]. Another consequence of the social stigma is a negative self-image, for example, due to verbal abuse in public areas. Other negative consequences include coerced sex, and being subjected to threats and violence.

Transgender communities in Nepal seem to be particularly vulnerable to social stigma and its consequences. Besides the above mentioned, they suffer from challenges such as discrimination, unemployment, violence and limited awareness and knowledge about sexual and gender minorities [29]. Furthermore, although it is legal in Nepal to have a gender and sexual minority identity, the public nuisance laws under the Public Offence Act [24] have been used to arrest members of gender and sexual minorities community [2, 30].

Although the difficulties faced by gender and sexual minorities are unlikely to leave this group of individuals unaffected, there is a great lack of research specifically focusing on mental health effects of such difficulties on these Nepalese minority groups [5]. Importantly, while recent reviews on this subject have been conducted in the USA or other English speaking and high-income countries [31], the question of negative health outcomes among gender and sexual minorities in low-income countries has only recently been raised on a sociological and psychosocial scientific level [3, 26]. The negative health outcomes vary from physical and mental health, to work and family-related social relations [25, 32–34]. A deeper knowledge and more comprehensive understanding of the negative mental health outcomes among sexual and gender minorities might help to identify possible interventions that may lead to a positive change in attitudes and, most importantly, to a better mental and physical health [3, 34–36].

The aim of this study was to analyze the impact of social and structural factors on depression and suicidality among MSM and TGW in Nepal.

## Methods

### Participants

Secondary cross-sectional data [37] were used in this study. Gender and sexual minorities are a large and diverse group of individuals. Therefore, we limited the study population in this study to MSM and TGW. Both MSM and TGW refer to several self-identified gender identities and sexual orientations. A list and definitions of these is available in Table 1.

**Table 1:** Definition of gender identities and sexual orientations of the study participants

Identity	Definition
MSM	
*Ta, panthi, gay, male*	Men assigned male at birth who are perceived as masculine, or “manly”, and who have a penetrative role when having sex with another man.
*Dohori*	“Both ways”. Men assigned male at birth who have no preferred role during sexual activity with another man.
TGW	
*Meti*	“A person who quenches a thirst”, i.e., has a receiving role in sexual activity. Transgender, feminine appearing and often cross-dressing person that was assigned male sex at birth.
*Meta*	“A man who quenches a thirst”.
*Kothi*	Southeast Asian counterpart for meti.
*Mougiya, mouga*	Local counterpart for meti in the Terai- region.
*Nachaniya*	Feminine appearing biological male who is a dancer.

*MSM* Men who have sex with men; *TGW* Transgender woman. For more information and references, see Additional file 1

#### Inclusion and exclusion criteria

For inclusion in the study the participants must have been assigned male at birth, aged 16 years or above and engaged in sexual relationship (oral and/or anal sex) with another biological male at least once within the past 12 months prior to the date of study. There were no exclusion criteria other than previous participation in the study.

#### Study area and data collection

The data was collected in April 2018 from 340 Nepalese MSM and TGW in eight small cities in the Terai Highway districts in Southern Nepal [37]. The eight districts where the study was carried out were: Jhapa, Morang, Sunsari, Nawalparasi, Rupandehi, Kapilbastu, Kailali and Kanchanpur. These districts share an open border with India. Besides the Terai region, similar data has previously been collected in Nepal’s two largest cities, Kathmandu (capital city) and Pokhara. Collecting data from the current districts provides a sample from smaller cities that all share an open border to India. This could potentially provide variations to the results compared with large city MSM- and TGW- population. The sample size was sufficient to detect a 15-% point difference according to a power analysis [37].

### Instruments and measures

#### Respondent-driven sampling

The participants were recruited by respondent-driven sampling (RDS). Generally, when using RDS, initial participants called “seeds” are identified [38]. In the current study there were eight seeds from various groups (regarding for example age, gender identity or sexual orientation) from each study district [37]. After participation in the study, each seed received three recruitment coupons that they gave to MSM and TGW within their social network. These participants, in turn, received recruitment coupons after completed participation. Participants reported their network size which was used to calculate RDS-weighted values.

The data was collected by face-to-face interviews using a semi-structured Integrated Biological and Behavioral Surveillance (IBBS) survey [37]. IBBS has been used in Nepal for HIV surveillance purposes for several years. The IBBS included questions about socio-demographic, structural, social and psychosocial factors. A list of the included factors is provided in Table 2. Information about sexual behavior and sexually transmitted diseases was also collected, but not used in this study. This was also the first time the IBBS data from Terai was used to assess psychosocial health problems among MSM and TGW.

**Table 2:** Descriptive statistics among 340 MSM and TGW in Nepal (RDS-weighted values)

Characteristics	MSM (***n*** = 201, 77%) *n* (%)	TGW (***n*** = 139, 23%) *n* (%)	Total *n* (%)
**Socio-demographic factors**			
Age			
≤ 30	133 (66)	67 (48)	200 (59)
> 30	68 (34)	72 (52)	140 (41)
Education			
Illiterate	16 (6)	19 (11)	35 (7)
Literate	168 (86)	104 (76)	272 (84)
Literate without formal education	17 (8)	16 (13)	33 (9)
Gender identity			
Third gender	38 (13)	99 (63)	137 (24)
Female	0 (0)	28 (20)	28 (4)
Male	163 (87)	12 (17)	175 (72)
Marital status			
Married	75 (28)	52 (34)	127 (29)
Not married	126 (72)	87 (66)	213 (71)
Main profession			
Student	60 (40)	3 (6)	63 (33)
Driver	9 (4)	0 (0)	9 (3)
Civil servant	6 (3)	0 (0)	6 (2)
Businessman	17 (6)	19 (11)	36 (7)
Private company staff	23 (8)	16 (12)	39 (11)
Laborer/wage labor	54 (28)	22 (21)	76 (24)
Artist	5 (2)	23 (11)	28 (5)
Farmer	11 (4)	11 (14)	22 (6)
Sex worker	8 (2)	34 (15)	42 (4)
Unemployed	8 (3)	11 (10)	19 (5)
Sex work past 12 months			
Yes	25 (6)	87 (51)	112 (16)
No	176 (94)	52 (49)	228 (84)
Income in NPR			
No income	29 (19)	6 (8)	35 (17)
< 10,000	31 (13)	28 (28)	59 (17)
10,000–19,999	64 (27)	53 (33)	117 (28)
20,000–29,999	18 (9)	22 (15)	40 (10)
≥ 30,000	24 (9)	23 (8)	47 (9)
**Structural factors**			
Cross-border movement for sexual activity			
Yes	45 (13)	42 (20)	87 (15)
No	156 (87)	97 (80)	253 (85)
*Experience of violence based on sexual orientation*			
Physical abuse			
Yes	6 (1)	19 (8)	25 (3)
No	195 (99)	120 (92)	315 (97)
Forced sex			
Yes	8 (1)	31 (1)	39 (4)
No	193 (99)	108 (99)	301 (96)
Cheated or threatened			
Yes	22 (7)	40 (7)	62 (9)
No	179 (93)	99 (93)	278 (91)
*Discrimination based on sexual orientation in different settings*			
At school			
Yes	32 (11)	52 (27)	84 (15)
No	169 (89)	87 (73)	256 (85)
Job hunting			
Yes	12 (2)	25 (12)	37 (4)
No	189 (98)	114 (88)	303 (96)
At work			
Yes	23 (6)	57 (29)	80 (11)
No	178 (94)	82 (71)	260 (89)
Getting housing			
Yes	5 (1)	27 (12)	32 (3)
No	196 (99)	112 (88)	308 (97)
Medical care			
Yes	7 (1)	97 (22)	49 (6)
No	194 (99)	42 (78)	291 (94)
Service in store or restaurant			
Yes	22 (6)	62 (33)	84 (12)
No	179 (94)	77 (67)	256 (88)
On the street or in a public setting			
Yes	28 (7)	87 (54)	115 (16)
No	173 (93)	52 (46)	225 (84)
From police or other security personnel			
Yes	24 (7)	58 (26)	82 (11)
No	177 (93)	81 (74)	258 (89)
Number of settings in past 12 months			
None	139 (81)	31 (38)	170 (71)
1 setting or more	62 (19)	108 (62)	170 (29)
**Social factors**			
Forced marriage with a female			
Yes	69 (26)	81 (53)	150 (32)
No	132 (74)	58 (47)	190 (68)
Evicted by family based on sexual orientation			
Yes	14 (2)	46 (22)	60 (7)
No	187 (98)	93 (78)	280 (93)
Family acceptance of sexual orientation			
Yes	37 (13)	55 (26)	92 (16)
No	164 (87)	84 (74)	248 (84)
**Psychosocial factors**			
Depression (CESD-R)			
Euthymic	81 (46)	31 (27)	112 (41)
Distress	63 (34)	45 (34)	108 (34)
Severe depression	57 (20)	63 (41)	120 (25)
Lifetime prevalence of suicidal thoughts			
Yes	20 (5)	58 (32)	78 (11)
No	181 (95)	81 (68)	262 (89)
Suicidal thoughts past 12 months			
Once or twice	12 (79)	14 (14)	26 (36)
A few times	4 (8)	23 (53)	27 (38)
Many times	3 (8)	21 (33)	24 (36)
No response	1 (5)	0 (0)	1 (2)
Lifetime prevalence of suicidal plans			
Yes	9 (26)	38 (51)	47 (43)
No	11 (74)	20 (49)	31 (57)
Lifetime prevalence of attempted suicide			
Yes	6 (15)	26 (28)	32 (23)
No	14 (85)	32 (72)	46 (77)
Alcohol consumption past 4 weeks			
None	86 (41)	73 (61)	159 (46)
At least once a week	109 (56)	54 (34)	163 (51)
Every day	6 (3)	12 (5)	18 (4)
Drug abuse past 12 months			
Yes	25 (8)	7 (1)	32 (7)
No	176 (92)	132 (99)	308 (93)

*MSM* Men who have sex with men; *TGW* Transgender woman; *RDS* Respondent-driven sampling; *n*: sub-sample size; *NPR* Nepalese Rupees; *CESD-R* Center for Epidemiologic Studies Scale Revised

The participants were first briefly screened to ensure that they met the inclusion criteria. After giving informed consent, participants were first interviewed to collect the behavioral data and then received pre-test counseling, before collection of biological specimen and health and STI checkup. Before finishing the survey process, they received test results as well as post-test counseling and referral for available services as per need [37].

#### Socio-demographic factors

Age was dichotomized to *≤30* (17–30 years old) and *> 30* (31–62 years old). Literate participants, with or without formal education were merged into one literate category, resulting in categories *literate* and *illiterate*. The *sexual orientation/gender identity* variable was merged into two categories with following division: *ta*, *man/mard*, *gay*, *panthi* to *MSM* and *meti/meta*, *Kothi*, *woman*, *transgender* to *TGW*. For more details regarding the terminology, see Table 1. *Sex work* was assessed by asking if a participant had had sex with a *male* or a *meti* for money in the past 12 months. Participants were also asked to report last month’s income in Nepalese rupees (NPR) and this was reported with categories < 10,000, 10,000–19,999, 20,000–29,999 and ≥ 30,000. Prior to logistic regression analysis the income was dichotomized using the mean income (15,000 NPR) as a cut-off.

#### Structural factors

Information on *cross-border movement for sexual activity* was collected by asking the participants whether they had crossed the open border to India for anal or oral sex the past 12 months (yes/no). *Experience of violence* was measured by yes/no- questions about *physical abuse*, *forced sex* and *cheated or threatened*.

#### Self-perceived discrimination

Self-perceived discrimination was measured using the self-report instrument Experiences of Discrimination (EOD) [39]. Firstly, participants were asked to answer whether or not they had experienced discrimination in different settings. These settings in the current study were *at school*, *getting hired or getting a job*, *at work*, *getting housing (renting or buying)*, *getting medical care*, *getting service in a store or restaurant*, *on a street or in a public setting (park)*, *from the police/other security personnel* [40]. Secondly, a summary was made of the number of settings where a participant had experienced discrimination (none, 1 setting or more).

#### Social factors

The social factors included questions about the participant’s family *forcing them to marry a female* (yes/no), *family evicting* the participant based on their sexual orientation (yes/no), and *family acceptance* of one’s sexual orientation by having at least one person within the family who the participant can openly talk to when it comes to their sexual orientation (yes/no).

#### Psychosocial factors

Aside from depression and suicidality below, other psychosocial factors include *Alcohol consumption in the past 4 weeks*, measured by adding the participants that never had drunk alcohol into the “none”- category thus creating three categories: *none*, *at least once a week* and *every day*. The same was done for *prevalence of drugs abuse in the past 12 months*. Participants reporting use of any drug were categorized as yes and the ones who never had used drugs were added to the category of participants who did not report specific drugs used in the past 12 months.

#### Depression

The primary outcome of interest, depression, was measured by Center of Epidemiological Studies Depression Scale Revised (CESD-R). The CESD-R consists of 20 self-reported questions [41]. The questions in the revised scale cover depressive symptoms defined by the fifth edition of Diagnostic and Statistical Manual (DSM-5), including *dysphoria*, *loss of interest*, *appetite*, *sleep*, *thinking/concentration*, *guilt*, *tiredness*, *agitation* and *suicidal ideation* [42]. In this study, the cutoff of 16 points of 60 possible was considered to correspond to a clinically significant depressive disorder [41]. Score for severe depression was set to 22 points, as used in previous studies [36]. The instrument has relatively high reliability and validity for research purposes [41, 43] and for depression assessment in both key and general population [44].

#### Suicidality

The secondary outcome of interest, suicidality, was measured by self-reported suicidal thoughts. All participants were asked whether they ever felt so low that they thought about committing suicide. For the ones that reported such thoughts, three additional questions were asked (“How often did you have any thought about ending your own life in last 12 months?”, “Have you ever made a plan to commit suicide?” and “Did you ever attempt suicide?”). However, the three additional questions were not included as outcomes of interest in the final analysis due to small sample size. The questions were extracted from DSM-5.

#### Statistical methods

We used IBM SPSS Statistics 25 (SPSS Inc., Chicago, IL, USA) in data analysis, and STATA 15 (StataCorp, College Station, Texas, USA) for RDS-weighted analysis. We performed a descriptive statistics analysis of the different variables, using frequencies and percentages for categorical variables, and mean and standard deviation for continuous variables. Bivariate and multivariable logistic regression analyses were then conducted to estimate crude odds ratio (OR) and adjusted odds ratio (AOR), respectively, and their 95% confidence intervals (CI). The dependent factors (outcomes of interest) in these analyses were *depression* and *suicidality*.

## Results

Most of the 340 participants were MSM (*n* = 201, 77%), and rest were TGW (*n* = 139, 23%). The mean age of the participants was 29 years (SD 10.12), ranging from 17 to 62 years. The majority of TGW identified as third gender (63%) whereas most of the MSM reported a male identity (87%). Most of the participants were literate (93%), but more of the MSM had a formal education (86%) than the TGW (76%). The most common main profession among all participants was student (33%), followed by wage labor (24%). Four percent of all participants listed sex work as their main profession, but 16% reported having earned money by selling sex during the past 12 months. The mean income per month was 15,000 NPR (SD 15900), which is equivalent to 134 U.S. dollars. For detailed descriptive statistics with RDS-weighted values, see Table 2.

Regarding the structural factors, 87 (15%) participants reported having crossed the open border to India for the purpose of sexual activity in the past year. Physical abuse based on sexual orientation was more common among TGW (*n* = 19; 8%) compared to MSM (*n* = 6; 1%). Both MSM and TGW reported the same rates of forced sex or being cheated or threatened based on sexual orientation (1 and 7%, respectively).

The mean number of settings where discrimination was experienced was 1.66 (SD 2.16). One-fifth of all MSM and three out of five TGW reported having suffered discrimination in more than one setting. TGW reported higher rates of discrimination compared to MSM in all eight settings. TGW experienced discrimination most commonly on streets or in public settings (*n* = 87; 54%) whereas MSM experienced most discrimination in school (*n* = 32; 11).

Regarding social factors, almost 1/3 of the participants’ families had forced, or attempted to force, them to marry a female. Specifically, over half of the TGW participants reported forced marriage compared to a quarter of MSM. Sixty participants (7%) had been evicted by their families based on sexual orientation. This rate was much higher among TGW compared to MSM (22 and 2%, respectively). Interestingly, family acceptance of sexual orientation was twice as common among TGW when compared to MSM (26% compared to 13%).

Generally, TGW reported more psychosocial health problems compared to MSM. Half of all participants drank alcohol at least once a week, but only 4% drank every day. Most of the participants who drank alcohol at least once a week were MSM (56% compared to 34% of TGW) whereas TGW had a greater occurrence of drinking every day (5% compared to 3% of MSM). Less than one in ten had used drugs during the past year, most of the drug users being MSM.

### Depression

**Fig. 1:**
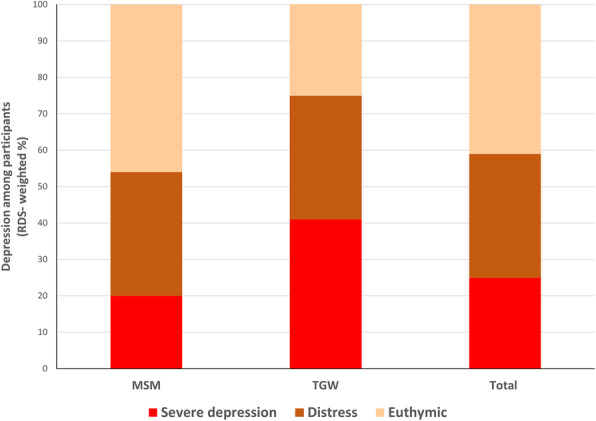
Depression among Nepalese MSM and TGW. RDS-weighted rates of depression (CESD-R) among MSM (*n* = 201), TGW (*n* = 139) and total study sample (*n* = 340). MSM: Men who have sex with men; TGW: Transgender woman; RDS: Respondent-driven sampling. CESD-R scores: Euthymic 0–15, Distress 16–21, Severe depression 22–37

Of all participants, 59% reached the cutoff score of 16 points on CESD-R. Equal rates of MSM and TGW, 34%, had a depression score between 16 and 21. More than twice as many TGW had severe depression compared to MSM (see Fig. 1 for RDS-weighted rates of depression).

### Suicidality

**Fig. 2:**
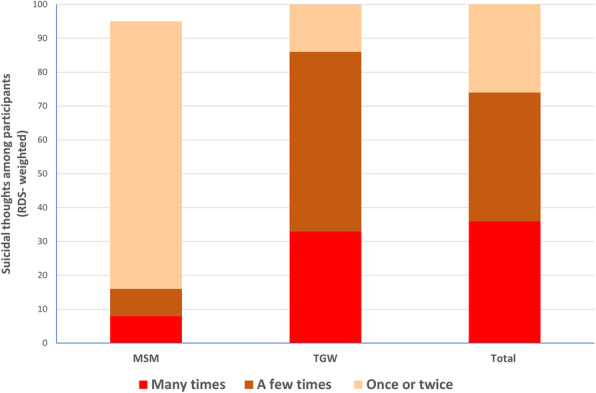
Suicidal thoughts among Nepalese MSM and TGW. RDS-weighted rates of suicidal thoughts in past 12 months among MSM (*n* = 19), TGW (*n* = 58) and total study sample (*n* = 77). MSM: Men who have sex with men; TGW: Transgender woman

Almost one-third of TGW had ever had suicidal thoughts compared to 5% among MSM. Of the participants reporting ever having had suicidal thoughts, over half of the TGW had made plans to commit suicide, and almost one third had attempted suicide. For comparison between MSM and TGW, see Fig. 2 for RDS-weighted rates of suicidal thoughts.

### Risk factors among MSM

As seen in Supplementary Table 1, eight different exposures were significant predictors of both depression and suicidality among Nepalese MSM. Two remained significant after including all exposures simultaneously in a multivariable logistic regression (see Table 3). Sex workers were more likely to report depression (AOR: 7.9; 95% CI: 3.4–18.2). The MSM who were evicted by their family based on sexual orientation were more likely to have suicidal thoughts (AOR: 6.2; 95% CI: 1.3–28.8).

**Table 3:** Effect of socio-demographic, structural, social and psychosocial factors on depression (CESD-R) and suicidality among 201 MSM

Variables	*n*	Crude OR	Adjusted OR
	(%)	(95% CI)	^b^(95% CI)
**Depression**			
Socio-demographic factors			
Sex worker			
No	43 (36)	ref	ref
Yes	77 (64)	**12.7 (5.9–27.1)**	**7.9 (3.4–18.2)**
**Suicidality**			
Social factors			
Evicted by family based on sexual orientation			
No	12 (60)	ref	ref
Yes	8 (40)	**19.4 (5.8–65.1)**	**6.2 (1.3–28.8)**

*CESD-R* Center for Epidemiologic Studies Scale Revised; *MSM* Men who have sex with men; *OR* Odds ratio; *n* sub-sample size; *CI* Confidence interval; *ref* reference category. See *Supplementary Table* *1* for all factors used in the bivariate and multivariable logistic regression

### Risk factors among TGW

Using bivariate logistic regression, we found several exposures that were significant predictors of depression and suicidality among Nepalese TGW (see Supplementary Table 2). As seen in Table 4, sex work (AOR: 6.5; 95% CI: 2.3–18.2) remained significant for depression after multivariable logistic regression. Participants who had been cheated and threatened (AOR: 3.9; 95% CI 1.2–12.5) and forced to marry a female (AOR: 2.2; 95% CI: 1.1–5.1) had higher odds of suicidal thought.

**Table 4:** Effect of socio-demographic, structural, social and psychosocial factors on depression (CESD-R) and suicidality among 139 TGW

Variables	*n*	Crude OR	Adjusted OR
	(%)	(95% CI)	^b^(95% CI)
**Depression**			
Socio-demographic factors			
Sex worker			
No	12 (11)	ref	ref
Yes	96 (89)	**11.1 (4.3–28.1)**	**6.5 (2.3–18.2)**
**Suicidality**			
Structural factors			
Cheated or threatened			
No	30 (52)	ref	ref
Yes	28 (48)	**5.3 (2.4–11.9)**	**3.9 (1.2–12.5)**
Social factors			
Forced marriage with a female			
No	17 (29)	ref	ref
Yes	41 (71)	**2.4 (1.2–5.1)**	**2.2 (1.1–5.1)**

*CESD-R* Center for Epidemiologic Studies Scale Revised; *TGW* Transgender woman; *OR* Odds ratio; *n*: sub-sample size; *CI* Confidence interval; *ref* reference category. See *Supplementary Table* *2* for all factors used in the bivariate and multivariable logistic regression

## Discussion

In this study, we aimed to analyze the impact of social and structural factors on depression and suicidality among MSM and TGW in Nepal. After multivariable analyses, several risk factors remained significant for depression and suicidality among MSM and TGW. We were also able to show that there are differences in several demographic factors between MSM and TGW in that TGW tended to suffer more discrimination.

### Strengths and limitations

This quantitative study had several strengths, including that the data were collected from a wide age span [17–62] and from municipalities that were both rural and included smaller cities (as opposed to large cities and metropolitan areas). In terms of methodology, the strength of this study is that recruiting participants was done with a validated sampling technique, RDS. The descriptive statistics are RDS- weighted thus providing more generalizable statistics. Besides RDS, even self- perceived discrimination, depression and suicidality were measured with standardized tools.

As for limitations in methodology, alcohol abuse was not measured with a standardized tool. Although RDS as a method has aimed to correct the flaws of its predecessor, that is, the snowball sampling method [38], it has yet to reach its full potential [45].

The cross-sectional nature of data collection for this study also leaves a question of how the psychosocial health issues developed over time in this sample [5]. Another limitation of the current study is that no comparison data from heterosexual cis-men or -women was collected. The findings of our study might be used to plan targeted interventions to improve mental health among gender and sexual minorities in Nepal. Although it is evident that the need for mental health services and prevention programs is great, there are no mental health services for gender and sexual minorities in the Terai region (Personal communication Ram Dev Tharu and Mamta Rai 1 Apr 2019). Furthermore, there is very limited mental health services available in the rest of the country (Personal communication Pinky Gurung 22 Mar 2019; Raju Thakali 25 Mar 2019). This leads to peer counseling often being the only option for help.

### Depression and suicidality

As many as 75% of TGW and 54% of MSM had clinically significant depression. Rates of depression are higher in our study compared to similar study in India where 42.7% of TGW and 35.3% of MSM had moderate to severe depression [46]. This study did however illustrate higher rates of depression among TGW compared to MSM. Another study on Indian MSM used CESD-R and found that 55% of participants met the cutoff criteria of 16 points, which is similar to our results [47]. Yet another study from India on psychiatric morbidities among TG showed that 31.2% suffered from current depression and 18.7% of dysthymia [48]. When it comes to suicidality, nearly 1/3 of TGW and 5% of MSM had ever had suicidal thoughts. Similar results in TGW, but higher rates among MSM have been reported by researchers in the U.S. [49, 50].

One significant factor contributing to suicidal ideation among TGW was forced marriage to a female. Not many published studies have found this association. Authors of a study of Kenyan MSM found lower depression scores among MSM who are married to a woman, and discuss the possibility that marriage to a woman could help MSM to conceal their sexual orientation [51]. They do not, however, mention whether or not the marriages are forced by family.

Nepal shares a long open border to India. Crossing this open border for sexual activities is a unique phenomenon that can hardly be expected to be found in many other regions. We found no articles where similar association was studied or found. To gain better understanding, we conducted interviews with individuals associated with gender and sexual minority rights movement in Nepal. In the Terai region, TGW sometimes sign contracts to provide sexual services to Nepali or Indian men, or to act as their partner for a certain period of time in exchange for money, accommodation or food (Personal communication Ram Dev Tharu and Mamta Rai 1 Apr 2019). Often these contracts are broken, possibly leaving TGW vulnerable with neither income nor accommodation. This could be one of the explanations to higher rates of depression and suicidality for individuals crossing the border (see Table 2), and even for sex workers and the ones reporting having been cheated or threatened. Threats and verbal abuse due to gender and sexual minority status, coming both from strangers and close ones, have been found to predict suicidality in other studies [49, 50].

Lack of social support was evident amongst many of the participants. Firstly, they could not openly talk about their own sexuality with their families, and secondly, many had been evicted by their family. Being evicted by family remained a significant risk factor for suicidality among MSM. Family rejection has in previous studies been associated with health problems such as suicide attempts, depression and use of drugs [52]. Evicted individuals in Nepal tended to move to larger cities and live under unstable housing conditions, for instance, with friends. Given that as many as half of the participants perceived discrimination in at least one setting, including the housing and working market, vulnerability for social problems among MSM and TGW is evident.

Unfortunately, this sometimes leaves sex work as the only solution for managing financially. Not only family problems [52] and lack of social support, but also being a sex worker increases the risk of having depression and displaying suicidal behavior. For both MSM and TGW, sex work remained as a significant factor for depression. Our findings support the analysis made in an Indian study, where sex work and depressive symptoms had positive correlation among MSM and TGW [53].

A larger proportion of the TGW experienced discrimination compared to MSM as seen in descriptive statistics (Table 2). A study from the U.S. found a high prevalence of attempted suicide among TG, with discrimination and depression among significant factors [54]. TGW tend to be more open about their sexuality compared to MSM who tend to be more afraid and attempt to hide their sexual identity [19], which could be one explanation to the higher rates of discrimination in our study.

In 2003, Meyer reviewed literature on how stress affects mental health among gender and sexual minorities [55]. Meyer summarized the findings of different (mostly American) studies and coined the minority stress theory. The theory posits that stressors such as adverse family factors and discrimination lead to mental health problems in an additive fashion. Our findings describe some of the possible stressors for Nepalese MSM and TGW. It follows that Meyer’s theory about higher prevalence of mental health problems among minorities, including gender and sexual minorities due to stress based on minority status, might be valid even in low-income, non-English speaking countries.

## Conclusions

Nepalese MSM and TGW suffer from a high degree of depression and suicidality. A factor increasing risk for depression among both MSM and TGW was sex work. Being evicted by family was associated with suicidality among MSM. For TGW, the associated factors for suicidality were being cheated or threatened and forced marriage to a female. Considering high burden of mental health issues among study population, it is vital to launch a mental health service program that reaches the gender and sexual minorities who already are suffering, as well as the minorities at risk to prevent and remedy mental health issues. However, mental and psychosocial health services and availability of mental health specialists are very limited in Nepal. It is also an utmost priority to generate evidence by implementing and evaluating effectiveness of peer-lead interventions to address high burden of mental health issues such as psychological distress among MSM and TG in Nepal.

## Supplementary Information

**Additional file 1.** Sexual orientations and gender identities of study participants.

**Additional file 2: Supplementary Table 1.** Effect of socio-demographic, structural, social and psychosocial factors on depression (CESD-R) and suicidality among 201 MSM. **Supplementary Table 2.** Effect of socio-demographic, structural, social and psychosocial factors on depression (CESD-R) and suicidality among 139 TGW.

## Data Availability

The datasets used and/or analyzed during the current study are available from the corresponding author on reasonable request.
