# Supplementary material for: Social and structural factors associated with depression and suicidality among men who have sex with men and transgender women in Nepal
Source: BMC Psychiatry. 2021 Sep 29;21:476. doi: 10.1186/s12888-021-03477-8 (PMC8479926; doi:10.1186/s12888-021-03477-8)
Supplement: Supplementary file 1 — Additional file 1. Sexual orientations and gender identities of study participants. [file 12888_2021_3477_MOESM1_ESM.docx]

**Title:** Social and structural factors associated with depression and suicidality among men who have sex with men and transgender women in Nepal

**Authors:** Sanna Storm, Keshab Deuba^§^, Rachana Shrestha, Lok Raj Pandey, Deepak Dahal, Madan Kumar Shrestha, Tara Nath Pokhrel, Gaetano Marrone

# Additional file 1. Sexual orientations and gender identities of study participants

*Men who have sex with men (MSM)*

An umbrella term for the non- transgender identities included in this study is MSM. The terms *ta,* *panthi*, *gay* and *male* all fall under this notion and describe identities for men assigned male at birth who are perceived as masculine, or “manly”, having a penetrative role when having sex with another man (1-4). Another identity that counts as MSM is *dohori*. It translates to “both ways”, describes men assigned male at birth who have no preferred role during sexual activity with another man (2).

*Transgender women (TGW)*

*Meti*, translating to “a person who quenches a thirst” (2, 5, 6), is a transgender, feminine appearing and often cross-dressing person that was assigned male sex at birth. “To quench a thirst” refers to having a receiving role in sexual activity (2) while appearing feminine refers to attributes such as long hair, wearing women’s clothes and feminizing or genital surgery (3). A similar identity in South Asia (including Nepal) is *kothi* (1, 2), and a local counterpart for the Terai- regions in southern Nepal is *mougiya* or *mouga*. A variant of *meti* is *meta*, meaning a person that rather identify as “a man who quenches a thirst” (2). Another TG- identity is nachaniya. It describes a feminine appearing biological male who is a dancer (Personal communication Pinky Gurung 22 Mar 2019).

**References**

1. Asia Pacific Forum of National Human Rights Institutions, United Nations Development Programme. Promoting and Protecting Human Rights in relation to Sexual Orientation, Gender Identity and Sex Characteristics: A Manual for National Human Rights Institutions [Internet]. Sydney: Asia Pacific Forum of National Human Rights Institutions;2016 [cited 2019 Feb 12]. Available from: http://www.asia‑pacific.undp.org/content/rbap/en/home/library/democratic_governance/hiv_aids/promoting-and-protecting-human-rights-in-relation-to-sexual-orie/
2. Boyce P, Pant S. Rapid Ethnography of Male to Male Sexuality and Sexual Health [Internet]. Kathmandu: Family Health International; 2001 [cited 2019 Mar 05]. Available from: https://www.who.int/hiv/topics/vct/sw_toolkit/ethnography_male_male_sexuality.pdf
3. Stief M. The Sexual Orientation and Gender Presentation of Hijra, Kothi, and Panthi in Mumbai, India. Arch Sex Behav. 2017 Jan;46(1):73-85.
4. Chakrapani V, Newman PA, Shunmugam M, Logie CH, Samuel M. Syndemics of depression, alcohol use, and victimisation, and their association with HIV- related sexual risk among men who have sex with men and transgender women in India. Glob Public Health. 2017 Feb;12(2):250-65.
5. Knight K. Bridges to Justice: Case Study of LGBTI Rights in Nepal [Internet]. New York: Astrea Lesbian Foundation for Justice; 2015 [cited 2019 Feb 14]. Available from: http://www.astraeafoundation.org/uploads/files/Astraea%20Nepal%20Case%20Study.pdf
6. United Nations Development Programme, Williams Institute. Surveying Nepal’s Sexual and Gender Minorities: An Inclusive Approach- Executive Summary [Internet]. Bangkok:UNDP Asia‑Pacific Regional Centre; 2014 [cited 2019 Feb 14]. Available from: https://www.undp.org/content/dam/rbap/docs/Research%20&%20Publications/hiv_aids/rbap-hhd-2014-surveying-nepals-sexual-and-gender-minorities-executive-summary.pdf
