# Supplementary material for: Social and structural factors associated with depression and suicidality among men who have sex with men and transgender women in Nepal
Source: BMC Psychiatry. 2021 Sep 29;21:476. doi: 10.1186/s12888-021-03477-8 (PMC8479926; doi:10.1186/s12888-021-03477-8)
Supplement: Supplementary file 2 — Additional file 2: Supplementary Table 1. Effect of socio-demographic, structural, social and psychosocial factors on depression (CESD-R) and suicidality among 201 MSM. Supplementary Table 2. Effect of socio-demographic, structural, social and psychosocial factors on depression (CESD-R) and suicidality among 139 TGW. [file 12888_2021_3477_MOESM2_ESM.docx]

**Supplementary table 1. Effect of socio-demographic, structural, social and psychosocial factors on depression (CESD-R) and suicidality among 201 MSM.**

| **Variables** | **Depression** | **Crude OR** | **Adjusted OR** | **Suicidality** | **Crude OR** | **Adjusted OR** |
| --- | --- | --- | --- | --- | --- | --- |
|  | *n* (%) | (95% CI) | ^b^(95% CI) | *n* (%) | (95% CI) | ^b^(95% CI) |
| **Socio-demographic factors** | | | | | | |
| Sex worker |  |  |  |  |  |  |
| No | 43 (36) | ref | ref | 5 (25) | ref | ref |
| Yes | 77 (64) | **12.7 (5.9-27.1)** | **7.9 (3.4-18.2)** | 15 (72) | **4.5 (1.5-13.1)** | - |
| **Structural factors** | | | |  |  |  |
| Cross-border movement for sexual activity | | | |  |  |  |
| No | 84 (70) | ref | ref | 11 (55) | ref | ref |
| Yes | 36 (30) | **3.4 (1.54-7.5)** | - | 9 (45) | **3.2 (1.2-8.5)** | - |
| *Experience of violence based on sexual orientation* | | | |  |  |  |
| Physical abuse |  |  |  |  |  |  |
| No | 114 (95) | ref | - | 16 (80) | ref | ref |
| Yes | 6 (5) | 5.7 (0.8-Inf.)* | - | 4 (20) | **22.3 (3.8-131.7)** | - |
| Sexual abuse |  |  |  |  |  |  |
| No | 112 (93) | ref** | - | 16 (80) | ref | ref |
| Yes | 8 (7) | **1.3 (1.03-1.84)** | - | 4 (20) | **11.1 (2.5-48.4)** | - |
| Cheated or threatened |  |  |  |  |  |  |
| No | 99 (82) | ref | ref | 11 (55) | ref | ref |
| Yes | 21 (18) | **16.9 (2.2-128.8)** | - | 9 (45) | **10.5 (3.7-30.1)** | - |
| Number of settings past 12 months | | | | | | |
| None | 67 (56) | ref | ref | 6 (30) | ref | ref |
| One or more | 53 (44) | **6.3 (2.8-13.8)** | - | 14 (70) | **6.4 (2.3-17.7)** | - |
| **Social factors** |  |  |  |  |  |  |
| Forced marriage with a female | | | | | | |
| No | 53 (44) | ref | ref | 6 (30) | ref | ref |
| Yes | 67 (59) | **3.2 (1.6-6.1)** | - | 14 (70) | **5.3 (1.9-14.6)** | - |
| Evicted by family based on sexual orientation | | | |  |  |  |
| No | 108 (90) | ref | ref | 12 (60) | ref | ref |
| Yes | 12 (10) | **4.3 (0.9-20.1)** | - | 8 (40) | **19.4 (5.8-65.1)** | **6.2 (1.3-28.8)** |

CESD-R: Center for Epidemiologic Studies Scale Revised; MSM: Men who have sex with men; OR: Odds ratio; *n*: sub-sample size; CI: Confidence interval; ref: reference category; **Exact logistic regression was performed due to one cell has 0 value; *** *removed sexual abuse from regression due to low number.*

**Supplementary table 2. Effect of socio-demographic, structural, social and psychosocial factors on depression (CESD-R) and suicidality among 139 TGW.**

| **Variables** | **Depression** | **Crude OR** | **Adjusted OR** | **Suicidality** | **Crude OR** | **Adjusted OR** |
| --- | --- | --- | --- | --- | --- | --- |
|  | *n* (%) | (95% CI) | ^b^(95% CI) | *n* (%) | (95% CI) | ^b^(95% CI) |
| **Socio-demographic factors** |  |  |  |  |  |  |
| Sex worker |  |  |  |  |  |  |
| No | 12 (11) | ref | ref | 8 (14) | ref | - |
| Yes | 96 (89) | **11.1 (4.3-28.1)** | **6.5 (2.3-18.2)** | 50 (86) | 2.3 (0.9-5.6) | - |
| **Structural factors** | | | |  |  |  |
| Cross-border movement for sexual activity | | | |  |  |  |
| No | 69 (64) | ref | ref | 34 (59) | ref | ref |
| Yes | 39 (36) | **5.2 (1.5-18.4)** | 2.9 (0.7-11.7) | 24 (41) | **2.4 (1.17-5.1)** | 1.4 (0.6-3.5) |
| *Experience of violence based on sexual orientation* | | | |  |  |  |
| Physical abuse |  |  |  |  |  |  |
| No | 90 (83) | ref | - | 45 (77) | ref | ref |
| Yes | 18 (17) | 6.0 (0.7-46.8) | - | 13 (23) | **3.6 (1.2-10.1)** | 1.1 (0.2-3.9) |
| Sexual abuse |  |  |  |  |  |  |
| No | 78 (72) | ref | ref | 37 (64) | ref | ref |
| Yes | 30 (28) | **11.5 (1.5-88.4)** | 1.3 (0.1-19.1) | 21 (36) | **4.0 (1.7-9.4)** | 1.1 (0.2-3.8) |
| Cheated or threatened |  |  |  |  |  |  |
| No | 69 (64) | ref | ref | 30 (52) | ref | ref |
| Yes | 39 (36) | **16.9 (2.2-129.1)** | 6.7 (0.5-89.4) | 28 (48) | **5.3 (2.4-11.9)** | **3.9 (1.2-12.5)** |
| Number of settings past 12 months | | | | |  |  |
| None | 20 (19) | ref | ref | 6 (10) | ref | ref |
| One or more | 88 (81) | **2.4 (1.0-5.8)** | 0.6 (0.2-1.9) | 52 (90) | **3.8 (1.4-10.1)** | 1.7 (0.5-5.2) |
| **Social factors** |  |  |  |  |  |  |
| Forced marriage with a female | | | | |  |  |
| No | 42 (39) | ref | - | 17 (29) | ref | ref |
| Yes | 66 (61) | 1.6 (0.7-3.7) | - | 41 (71) | **2.4 (1.2-5.1)** | **2.2 (1.1-5.1)** |
| Evicted by family based on sexual orientation | | | |  |  |  |
| No | 69 (64) | ref | - | 33 (57) | ref | ref |
| Yes | 39 (36) | 1.9 (0.7-4.9) | - | 25 (43) | **2.1 (1.1-4.4)** | 1.1 (0.4-2.5) |

CESD-R: Center for Epidemiologic Studies Scale Revised; TGW: Transgender woman; OR: Odds ratio; *n*: sub-sample size; CI: Confidence interval; ref: reference category. **Exact logistic regression was performed due to one cell has 0 value.*
